# Aptamers: Novel Molecules as Diagnostic Markers in Bacterial and Viral Infections?

**DOI:** 10.1155/2013/731516

**Published:** 2013-09-05

**Authors:** Flávia M. Zimbres, Attila Tárnok, Henning Ulrich, Carsten Wrenger

**Affiliations:** ^1^Unit for Drug Discovery, Department of Parasitology, Institute of Biomedical Science, University of São Paulo, Avenida Professor Lineu Prestes 1374, 05508-000 São Paulo, SP, Brazil; ^2^Department of Pediatric Cardiology, Heart Centre Leipzig, Translational Centre for Regenerative Medicine (TRM), University of Leipzig, Strümpellstraße 39, 04289 Leipzig, Germany; ^3^Department of Biochemistry, Institute of Chemistry, University of São Paulo, Avenida Professor Lineu Prestes 748, 05508-900 São Paulo, SP, Brazil

## Abstract

Worldwide the entire human population is at risk of infectious diseases of which a high degree is caused by pathogenic protozoans, worms, bacteria, and virus infections. Moreover the current medications against pathogenic agents are losing their efficacy due to increasing and even further spreading drug resistance. Therefore, there is an urgent need to discover novel diagnostic as well as therapeutic tools against infectious agents. In view of that, the Systematic Evolution of Ligands by Exponential Enrichment (SELEX) represents a powerful technology to target selectively pathogenic factors as well as entire bacteria or viruses. SELEX uses a large combinatorial oligonucleic acid library (DNA or RNA) which is processed a by high-flux *in vitro* screen of iterative cycles. The selected ligands, termed aptamers, are characterized by high specificity and affinity to their target molecule, which are already exploited in diagnostic and therapeutic applications. In this minireview we will discuss the current status of the SELEX technique applied on bacterial and viral pathogens.

## 1. Introduction

**Figure 1:**
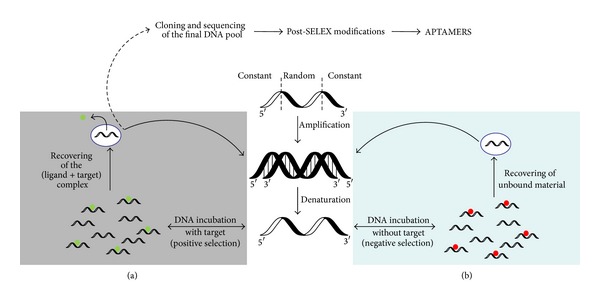
Schematic illustration of the SELEX methodology. The SELEX technique uses a large combinatorial oligonucleic acid library (DNA or RNA) consisting of an inner random region flanked by two constant regions. In the following, attention has solely been drawn on the development of DNA aptamers. The DNA library consisting of partially randomised DNA sequences (inner random region flanked on both sites by constant sequences) is amplified by conventional PCR. The derived double-stranded DNA is denatured and separated into single-stranded DNA by gel electrophoresis, the single-stranded DNA isolated and subsequently incubated with the respective target molecules ((a), positive selection). After incubation step, the formed target-aptamer-complex is separated from nonbinding aptamers and applied to PCR for amplification of the target-bound aptamers (grey panel). Eventually, (b) negatives cycles (blue panel) are also carried out to remove aptamers which bind unspecifically or not to the desired target molecules. As a resulting consequence, the unbound aptamers are recovered, amplified via PCR, and applied in the next (positive) selection cycle. Subsequently, from final selected library aptamer sequences are identified and aligned for the verification of consensus sequence motifs. If required post-SELEX modifications such as truncations, stabilizations, and covalent attachment of fluorescence reporters can be applied to optimize aptamers for any desired purpose.

**Figure 2:**
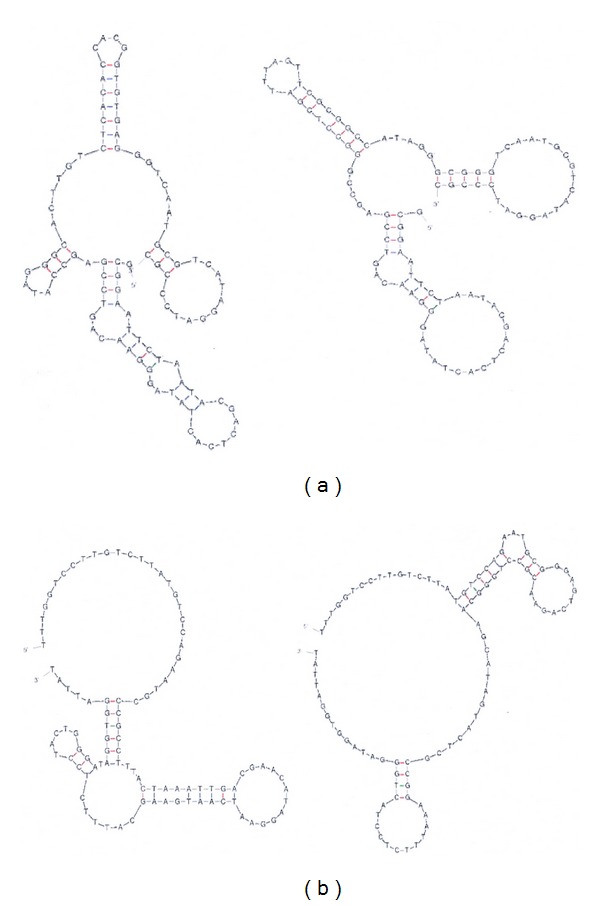
Predicted folding of selected single-stranded DNA aptamers. Stem-loop structures of aptamers play a fundamental role in target molecule binding. Respective consensus sequences of aptamers binding to (a) Hepatitis C virus (HCV) E2 glycoprotein on the cell surface [24] and (b) S. Typhimurium outer membrane proteins (OMPs) [25] were analyzed using the MFold programme [26] for secondary structure predictions.

Due to the continuously rising number of the population as well as the emergence of resistances of human pathogenic organisms against the current treatment, there is an urgent need to develop novel diagnostic and—even further—therapeutic tools to deal with the upcoming problems in the near future. Not only the development of new tools is mandatory but also—and becoming an even more prominent issue—the commercial value in terms of their costs. In this sense the discovery of novel technologies and moreover their subsequent applications have become important in laboratory and field studies. As an example, innovations in high-throughput single cell analysis for diagnostic purposes but also for functional analysis and drug discovery are today available in order to analyze pathogenic organisms such as the viruses Hepatitis C [1], influenza [2], or HIV [3] as well as bacteria like *Mycobacterium tuberculosis* [4] or *Staphylococcus aureus* [5]. The SELEX technique (Systematic Evolution of Ligands by Exponential Enrichment) was originally discovered by Gold and Szostak [6, 7]. They were using reiterative *in vitro* selection for high-affinity oligonucleotide ligands (aptamers) against almost any kind of molecules which is of biological or therapeutic interest (for illustration see Figure 1). RNA and DNA aptamers recognize their targets with high specificity and affinity. These high-affinity ligands can be developed against almost any target through iterative cycles of *in vitro* screening of a combinatorial oligonucleotide library for target binding followed by their PCR amplification. SELEX procedures are characterised by employing random oligonucleotide libraries of up to 10^12^–10^15^ different nucleic acid molecules, revealing an at least equivalent number of secondary and tertiary structures of their respective single-stranded nucleic acid molecules [8]. After their first round of *in vitro* selection against a chosen target molecule, the eluted aptamers are amplified by PCR procedures to restore the DNA library for the next SELEX cycle. The number of cycles depends mainly on the affinity of the aptamer-target interactions as well as on the stringency imposed to each round of selection. After several rounds of iterative SELEX cycles, the aptamers are selected via their target molecules (positive selection step) (Figure 1). In case of not using highly pure target molecules, the library needs to be exposed to different or contaminating molecules for counterselection in order to discard these molecules (negative selection step) (Figure 1). Subsequently, the final selected library of high affinity aptamers are cloned and sequenced to identify respective consensus motifs, which are responsible for the secondary/tertiary structures interacting with the target molecule [9]. These structures comprise a variety of different conformations which include—among others—stem-loop structures. Comparisons between DNA or RNA stem-loops suggest that the structure of the DNA molecules can be slightly less stable. On the other hand—due the lacking ribose 2′-hydroxyl group—DNA is characterised by a greater flexibility and, consequently, leading to a higher diversity of structural conformations [10]. In contrast to DNA aptamers, RNA aptamers require reverse transcription prior amplification by PCR and in terms of their stability they might need modifications such as substitution of the 2′-OH group of ribose by 2′-amino, 2′-fluoride, or O-methylene functions, to prolong their half-life in biological fluids [11, 12]. Unlike RNA aptamers which are obliged to modifications in order to prevent RNAse accessibility, DNA aptamers do not need any modifications for their stability in various applications. Moreover, the latter one can be easily modified for the attachment of reporter molecules such as fluorescence dyes or biotin [13]. Those biotinylated aptamers can subsequently be applied in pull-down experiments using streptavidin-coated magnetic beads [14, 15] or to visualise targets using fluorescent reporters [16]. The binding properties of an aptamer are dictated by its sequence and the deriving folding into stem-loop structures (Figure 2) [13, 17]. Aptamer-target complexes often reveal low dissociation constants that range from nanomolar to picomolar levels [9, 18], which is comparable to those of monoclonal antibodies.

Moreover, aptamers are capable of distinguishing between protein isoforms [19] as well as different conformational forms of the same protein [20]. Furthermore, aptamers can be denatured and renatured by changing the temperature. Additionally they are generated by an iterative *in vitro *instead of an *in vivo* process as it is the case for animal-derived antibodies [16]. 

In the near future oligonucleotide-based high-affinity aptamers are expected to substitute antibodies in many applications, mostly due to their stability, nonpeptide character, and their independency from animal resources. Moreover, the beneficial characteristics of aptamers have already been exploited in the fight against infectious diseases as outlined below. 

## 2. Overview of Aptamer Applications in Human Infection Diseases

Infectious diseases affect almost the entire mankind as well as animals. Furthermore, increasing levels of pathogen resistance against available drugs aggravate the state of health worldwide, particularly in developing countries, where infectious diseases are responsible for a high level of mortality and morbidity [21]. Moreover, the continuously growing population combined with the ongoing drug resistance forces scientific research to discover novel diagnostic tools as well as chemotherapeutics to deal with the future problems caused by pathogenic agents. Here, we will focus on the status of SELEX applications for combating bacterial and viral human infections.

## 3. Aptamers and Bacterial Infections

Tuberculosis, along with malaria and HIV, is a major public health problem affecting millions of people worldwide. Currently, only one vaccine against tuberculosis is existing which is the attenuated strain of *Mycobacterium bovis* “BCG—bacillus Calmette-Guėrin”; however, the vaccine has only limited efficacy in tuberculosis-endemic regions [22]. In 2007 Chen et al. [23] isolated an aptamer entitled NK2 with high affinity and specificity against membrane proteins that are present on the surface of the virulent *M. tuberculosis* stain H37Rv while not existing on BCG. Furthermore, the binding of the NK2 aptamer to H37Rv led CD4+ T cells to produce an increased level of IFN-*γ*, which is known to be a protective cytokine against *M. tuberculosis* infections. Analysis of the spleen of mice treated with the NK2 aptamer showed a lower bacterial number as the control [23]. These results underline the potency of the NK2 aptamer as an antimycobacterial agent (Table 1).

Another common bacterial infection is caused by Salmonellae which are significantly involved in food-borne illness. About 25% of all food-borne diarrhoea patients need hospitalisation. A major problem is salmonellosis caused by multidrug-resistant (MDR) strains such as *Salmonella enterica *serovar Typhimurium DT104 or *S. enterica *serovar Newport [27]. Salmonellae are also important bacterial pathogens in food animal species including cows [28], pigs [29], chickens [30], and turkeys [31]. In order to evaluate the potential of aptamers against *S. enterica *serovar Typhimurium multidrug-resistant (MDR) strains, the SELEX method using DNA aptamers was applied to the bacterial outer membrane proteins (OMPs) by performing counter-selection against *Escherichia coli* OMPs and lipopolysaccharides (LPS) (negative selection step). Subsequently aptamers were identified which selectively interact only with the bacterial OMPs [25] (Table 1).

It is known that the invasion of IVB piliated *S*. *enterica *serovar Typhimurium A21-6 into human THP-1 monocytic cells reveals higher efficacy than that of a nonpiliated *S*. *enterica *serovar Typhimurium mutant strain. Consequently, approaches have been undertaken to interfere with the formation and accessibility of the type IVB pili. Thus, the SELEX technique was applied and single-stranded RNA aptamers were discovered that selectively bind to the pili and thereby hamper the infection of human THP-1 cells [32]. Analysis of the consensus sequence identified a stem-loop structure, which might be the possible binding site of the aptamer [32]. In summary, these RNA aptamers are considered as a novel agent for blocking bacterial pathogenesis (Table 1).

Bacteria such as *S. aureus* or *E. coli* are naturally present in humans. For example, *S. aureus* colonises permanently about 20% of healthy adults and up to 50% transiently. Its pathogenicity plays an important role in nosocomial infections affecting immunosuppressed patients. This pathogenic agent is responsible for a wide spectrum of diseases, which includes life-threatening conditions like pneumonia or endocarditis [42], and emerges as a major human pathogen due to methicillin-resistant *S. aureus* (MRSA) strains. These bacteria are known to produce potent protein toxins and to express cell-surface proteins that can bind to antibodies and thereby inactivate their function [43]. It is well established that bacteria can express different sets of molecules to precisely control their proliferation at different growth states [44, 45]. They can even undergo antigenic variations to escape the immune response of its host [46, 47]. In order to deal with these bacterial mechanisms, a whole set of specific markers have been developed by Cao and coworkers [33]. They obtained a panel of single-stranded DNA aptamers with high specificity and sensitivity against *S. aureus*. The derived aptamers were grouped into different families on the basis of sequence homology as well as similarity in their predicted secondary structure. Subsequently five aptamers were identified that recognised different molecular targets, and a combination of them had a much better effect than the application of individual aptamers in detecting different *S. aureus* strains. These results clearly demonstrate that a set of aptamers specific to one bacterium can be used as a highly selective diagnostic tool or even potentially to block the growth cycle of the pathogen [33].

## 4. Aptamers in Viral Infections

Hepatitis C is an infectious disease caused by the hepatitis C virus (HCV), which has infected about 3% of the world's population and induces in 80% of the infected people dysfunction of the liver, like cirrhosis. Presently, there is no efficient vaccine available, and the treatment of the infectious disease relies on the use of alpha interferon (IFN-*α*) alone or in combination with ribavirin [48–50]. However, the rate of success is limited, and such treatments are expensive and bear the risk of serious side effects [48, 49, 51]. In order to discover new modes of detection and selective interference with the proliferation of the virus, the alive Cell Surface-Systematic Evolution of Ligands by Exponential Enrichment (CS-SELEX) technique has been developed and subsequently used for selection of single-stranded DNA aptamers directed to the HCV envelope surface glycoprotein E2 [24]. A single-stranded DNA aptamer that specifically binds to the HCV-E2 envelope glycoprotein was named ZE2. It is believed that the ZE2 aptamer competitively inhibits the HCV-E2 envelope glycoprotein by binding to CD81, an important HCV receptor, and significantly blocks HCV cell culture infection of human hepatocytes. Thereby, the ZE2 aptamer emphasizes its potency to act as a possible novel diagnostic and therapeutic candidate in HCV infections. 

Avian influenza virus (AIV) H5N1, also known as “bird flu,” is a type A influenza virus, responsible for major epidemics and pandemics. Therefore attention has been drawn—besides the use of other therapeutic tools—on the selection of aptamers against the H5N1 virus. Wang et al. [34] started with a single-stranded DNA library and performed the first rounds of selection cycles using purified hemagglutinin (HA) from AIV H5N1, and afterwards the entire H5N1 virus was used as a target. After various SELEX rounds of positive target selection and clearance of selected pools of oligonucleotides binding to nontarget AIV subtypes (H5N2, H5N3, H5N9, H7N2, H2N2, and H9N2), aptamers were cloned and sequenced. The found consensus sequences were analysed for predicted secondary structures, which led to the identification of hairpin loops and bulge loops that are suggested to play an important role in target binding. By using surface plasmon resonance, one aptamer was identified possessing a high binding capability with a dissociation constant of less than 5 nM to the H5N1 virus.

Rabies is a zoonotic disease, which is caused by the rabies virus (RABV), and is transmitted through exposure to infected saliva during either a bite or direct contact with mucosal tissues. Infection with this virus results in an acute fatal encephalitis, leading to coma and death. This disease affects many warm-blooded mammals. Currently, no approved therapy is available once the clinical signs have appeared. By being a fatal disease in all instances, there is a strong incentive to develop a cheap and effective drug. In this sense aptamers were selected against RABV-infected cells using the Cell-SELEX technique [35]. These aptamers were subsequently applied to viral titre assays, which clearly demonstrated an inhibition of RABV-infected cell, while an inhibition of the canine distemper virus or canine parvovirus was not observed. Furthermore, in *in vivo* tests aptamers could protect mice to a certain extent from RABV infection. Interestingly, the selected aptamers were of protective nature because when mice were inoculated with aptamers before inoculation with CVS-11, only about 15% of the animals died, whereas almost no mice survived when aptamers were used for treatment [35].

Such as other pathogens, HIV is also characterised by the appearance of drug-resistant viruses. About 30 years ago, it was discovered that HIV contains several small RNA sequences or regions which can specifically bind to viral or cellular proteins with high affinity. Functional studies indicated that these viral RNA-protein complexes could be exploited in therapeutic approaches as demonstrated for small HIV RNA regions, termed TAR, that could be used to inhibit HIV virus replication in cellular models [52]. The envelope glycoprotein gp120 of the HIV-1 virus plays a fundamental role during infection of CD4-positive cells. Cell fusion is initiated by the interaction between gp120 and CD4 (Table 1). In view of this mechanism, RNA aptamers were generated, which bind selectively to the gp120 glycoprotein [53, 54]. In a humanized mouse model where HIV-1 replication and T-cell depletion mimic the human situation, Neff and colleagues [36] found that the anti-gp120 aptamer suppressed HIV-1 replication and prevented thereby the viral-mediated T-cell reduction. 

The SELEX technique was also applied to the viral reverse transcriptase, which is essential for replication, and thereby RNA and DNA aptamers were identified that bound to multiple epitopes of the viral reverse transcriptase with high affinity and specificity [37, 38, 55]. Among those aptamers, pseudoknot RNAs have received attention which bind to the HIV-1 reverse transcriptase at a nanomolar level [39–41], abolish its catalytic activity [38], and inhibit HIV replication in cell culture [40, 56, 57]. These results clearly emphasize that aptamers binding to the HIV-1 reverse transcriptase are promising novel tools to be used in therapeutic interventions. 

## 5. Conclusion

As outlined above bacterial and viral infection diseases are a major threat to humans. Considering that the current therapeutics are losing their efficacy, there is an urgent need to discover and develop novel medication and rapid diagnostic to deal with these diseases. In this sense the SELEX technology by using aptamers provides a powerful tool not only to identify novel diagnostic markers but also to interfere with the proliferation of these human pathogens [16]. The aptamer technique is already subject to a variety of clinical trials in human-related diseases [58]. A VEGF165-binding aptamer, Macugen, was recently commercialised as an antiangiogenic therapeutic agent for neovascular age-related macular eye disease [59, 60]. Macugen has been a breakthrough in the therapeutic use of aptamers and encourages the development of further aptamers against infectious disease targets as outlined in this minireview and summarized in Table 1.

## Figures and Tables

**Table 1:** Summary of aptamers against bacterial and viral human pathogens.

Aptamers	Type of aptamer	Organism	Target	Reference
NK2	DNA aptamer	*Mycobacterium tuberculosis* (strain H37Rv)	Membrane proteins	[23]
33	DNA aptamer	*Salmonella enterica *serovar Typhimurium	Outer membrane proteins (OMPs)	[25]
S-PS8.4	RNA aptamer	*Salmonella enterica *serovar Typhimurium	Type IVB pili	[32]
SA20, SA23, SA31, SA34 and SA43	DNA aptamer	*Staphylococcus aureus* (strain MRSA)	Whole bacteria	[33]
ZE2	DNA aptamer	Hepatitis C virus	HCV envelope surface glycoprotein E2	[24]
Sequence (1), aptamer sequence (2), and aptamer sequence (3)	DNA aptamer	Avian influenza virus H5N1	Hemagglutinin	[34]
FO21 and FO24	DNA aptamer	Rabies virus (RABV)	RABV-infected BHK-21 cells	[35]
A-1 and Ch A-1	RNA aptamer	Human immunodeficiency virus type 1 (HIV-1)	gp120	[36]
RT1t49	DNA aptamer	Human immunodeficiency virus type 1 (HIV-1)	Viral reverse transcriptase	[37]
70.5, 70.8, 80.55, 80.93 and T1.1	RNA aptamer			[38]
TPK isolates, TPK-like isolates and non-TPK isolates	RNA aptamer			[39]
70.8, 13, 70.15, 80.55, 65, 70.28, 70.28t34 and 1.1	RNA aptamer			[40]
1.1 and 1.3a	RNA aptamer			[41]
